# PBJ ranks higher, despite publishing more original articles, very few editorial materials and offers free global access

**DOI:** 10.1111/pbi.13313

**Published:** 2019-12-18

**Authors:** Henry Daniell

**Affiliations:** ^1^ Department of Basic and Translational Sciences, School of Dental medicine University of Pennsylvania Philadelphia PA USA

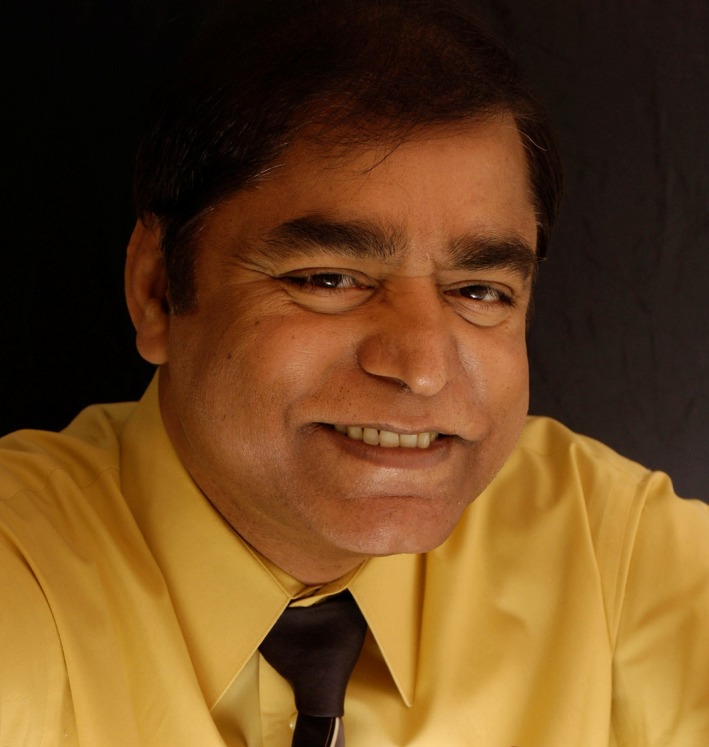

Welcome to the first issue of the eighteenth volume of the *Plant Biotechnology Journal*. PBJ has successfully transitioned from a subscription‐based journal to number one open‐access plant science journal. This transition has resulted in enhanced free global access to all our readers, although the open‐access fee burden is borne by the authors.

Scopus CiteScore ranks *PBJ* first among 320 Agronomy and Crop Science journals. Excluding review journals in plant sciences, CiteScore ranks *PBJ* third (6.88) behind New Phytologist (7.17) and Molecular Plant (7.15) but above Nature Plants (6.65) and the *Plant Cell* (6.88). CiteScore metrics address some of the major limitations of the Impact Factor, which excludes certain article types (Letters, Commentaries, Editorials) that decrease the number of citable items but contribute citations. *PBJ* receives the lowest number of citations from editorial items (21 citations, 1.4%) that are not counted towards the impact factor, when compared to other plant science journals (e.g. 430 citations, 29% of *Molecular Plant*).

PBJ increased Impact Factor from 6.32 to 6.84 this year, ranking higher than Plant Physiology (6.30) and the Plant Journal (5.72). PBJ ranks #5 among plant science journals publishing original research articles. Based on my current evaluation of citations, PBJ is likely to reach >8.0 Impact Factor and CiteScore in 2019 because of exceptionally high citations, with ~15% of highly cited articles that are ranked within the top one percentile of articles published in plant and animal sciences. Irrespective of the method of citation analysis, *PBJ* is building on the strength of high impact original inventions reported by our authors and critically evaluated by our editors/reviewers. Therefore, I convey my greatest appreciation to our authors for submitting their best research and our editors/reviewers for their critical evaluations. I look forward to your continued support in 2020.

Since I started as the Editor in Chief in 2012, submission of manuscripts has almost tripled, despite transition to an open‐access journal few years ago. PBJ received a record number of manuscripts from 58 countries representing all continents around the globe in 2019, especially with more submissions from Asia, Europe and Australia. I am deeply grateful for thousands of critical evaluations received from reviewers and their generous time commitment. Timely reviews have significantly decreased the average turnaround time, rewarding our authors with timely decisions on their submissions. Considering *PBJ* is still a very young plant science and biotechnology journal, these are very impressive accomplishments.

PBJ has increased social media activities in 2019 under the exceptional leadership of Ms. Hannah Qualtrough, Senior Marketing Manager at Wiley. Professor Shuangxia Jin (PBJ Associate Editor, Huazhong Agricultural University, China) has successfully launched the PBJ Wechat account on 1 March 2019 publishing 1,123 news articles including 364 original articles written by his students and 759 articles cited from other social media sources. This has resulted in 18,150 Wechat accounts of plant scientists, with 1,341,024 hits from 806,283 computers, 5967 hits/day and 1,750 hits per PBJ news release. In order to increase such global outreach, PBJ will start publishing foreign language abstracts to enhance outreach in their countries through the social media. Therefore, authors are encouraged to submit abstracts in English and their local languages, which will be edited by PBJ Associate Editors or editorial board members with representation from all continents. I request that authors provide Twitter titles at the time of manuscript submission. Posts from PBJ Twitter account had 32,515 impressions. In addition, I encourage authors to share news releases on their articles with the *PBJ* editorial office so that they are included in Wiley Plant Science tweets @PlantSciNews, which currently has >15,000 followers.

I convey my sincere thanks for the excellent service offered by all our Associate Editors who have served more than ten years (Profs. Dominique Michaud, Malcolm Campbell) or in the past few years (Profs. Neal Stewart, Dave Edwards, Stephen Streatfield, Johnathan Napier, Xiao‐Ya Chen, Nicola Patron, Martin Parry, Rajeev Varshney, Kan Wang) or joined recently (Drs. Caixia Gao, Zuhua He, François Belzile, Shuangxia Jin, Jihong Liu‐Clarke, Marco Maccaferri). In 2020, PBJ website will feature brief biographies of editors and videos to introduce their research areas to our readers and authors.

Without the outstanding leadership of Mr. Jim Ruddock – Managing Editor and Ms. Rosie Trice – Senior Publishing Manager and Ms. Lauren Dawson Editorial Assistant at Wiley, Oxford, PBJ will not be able to function and I convey my deepest appreciation. I thank Ms. Maricar Dumlao – *PBJ* production editor at Wiley, Manila for efficient and timely production and release of PBJ every month.

*PBJ* is now compatible with mobile platforms, tablets, iPads and iPhones and offers several new options to evaluate the short‐ and long‐term impacts of published articles, including Altmetric scores, article readership and citations. I encourage all readers to visit the journal homepage (http://onlinelibrary.wiley.com/journal/10.1111/%2528ISSN%25291467-7652) to take advantage of open access, keep up to date with latest developments and to sign up for our automated e‐alerts in order to receive emailed notifications when new issues or Early View articles are published. Please note that readers should ‘opt‐in’ to receive e‐alerts, by visiting the journal homepage and registering at the ‘Get Content Alerts’ area.

*PBJ* management has approved my request to waive or reduce open‐access fees for manuscripts recommended for publication from authors who do not have adequate funding for publications. Therefore, I am fully committed to advancing *PBJ*’s mission of publishing high‐quality manuscripts with free global access and look forward to your continued support in 2020.

